# Purtscher-Like Retinopathy in a Patient with COVID-19

**DOI:** 10.1155/2021/6661541

**Published:** 2021-03-20

**Authors:** Alexander R. Bottini, Sean Steinmetz, Kevin J. Blinder, Gaurav K. Shah

**Affiliations:** The Retina Institute, St. Louis, MO, USA

## Abstract

The emerging literature on the novel coronavirus pandemic has reported several cases of varied retinal findings in patients with COVID-19. We report the case of a 59-year-old male who presented with complaint of bilateral blurry vision following hospital discharge after prolonged hospitalization for severe COVID-19 illness. On ocular exam, the patient demonstrated bilateral cotton wool spots localized to the posterior pole of each eye. Multimodal imaging demonstrated findings consistent with retinal nerve fiber layer infarcts in the areas of the cotton wool spots. Exam and imaging of our patient were most consistent with a Purtscher-like retinopathy. We suggest that as ophthalmologists care for increasing numbers of patients recuperating from COVID-19, they monitor for microangiopathic changes similar to those in our patient.

## 1. Introduction

Purtscher's retinopathy—featuring cotton wool spots, retinal hemorrhages, and inner retinal opacification (Purtscher flecken)—is classically described following significant traumatic injury [1, 2]. Purtscher-like retinopathy describes similar retinal findings associated with a variety of other conditions including pancreatitis, thrombotic thrombocytopenia purpura, and hemolytic uremic syndrome [1, 2]. We present the case of a patient recovering from severe COVID-19 illness requiring prolonged hospitalization who presented with a retinopathy most consistent with Purtscher-like retinopathy.

## 2. Case Report

A 59-year-old white male presented with complaint of bilateral blurry vision. The patient had noticed a subjective change in visual acuity in the preceding two weeks, which followed his hospital discharge after a prolonged hospitalization for COVID-19.

Past medical history included coronary artery disease, insulin-dependent diabetes mellitus, hypertension, and hyperlipidemia. Six weeks prior to ophthalmic evaluation, the patient presented to the emergency department with fever, cough, and progressive dyspnea. SARS-CoV-2 testing was positive, and chest radiographs demonstrated bilateral infiltrates consistent with COVID-19 pneumonia. The patient's ability to oxygenate quickly deteriorated, and he was admitted to the intensive care unit with acute hypoxemic respiratory failure and intubated shortly thereafter.

The patient's month-long hospital course was complicated by deterioration of his respiratory status, requiring extracorporeal membrane oxygenation for eleven days. His treatment also included convalescent plasma and remdesivir. The patient also developed encephalopathy and coagulopathy requiring full-dose anticoagulation. Blood pressure was labile, and at times, multiple antihypertensive medications were required. His blood glucose levels were well controlled throughout his hospitalization.

**Figure 1:**
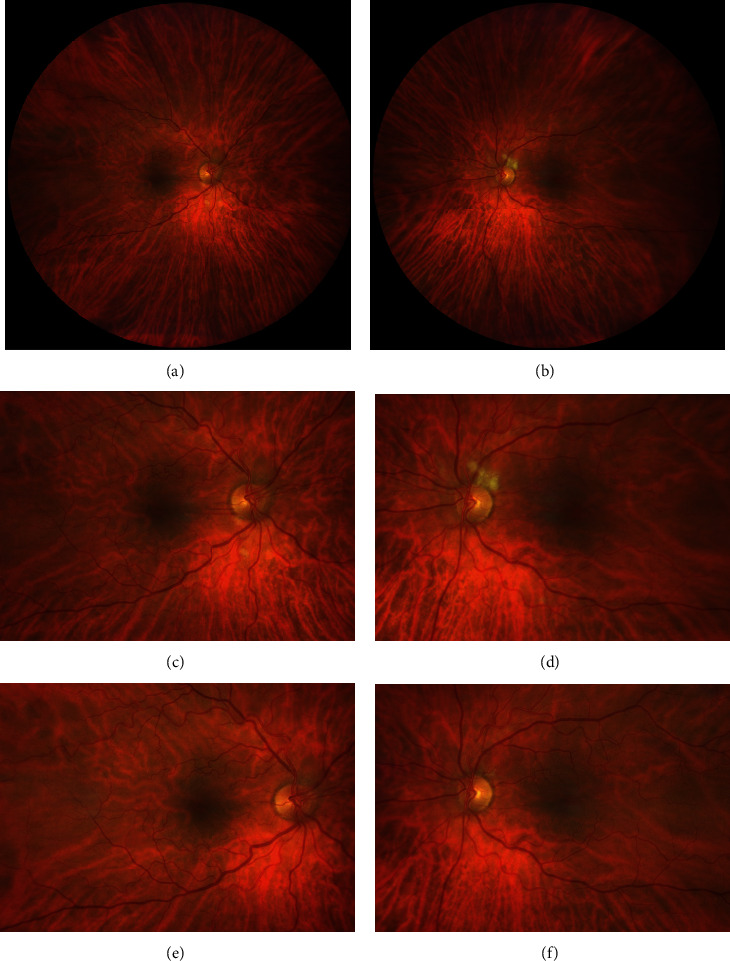
Fundus photos demonstrating cotton wool spots in the posterior pole of each eye at presentation (a–d) and their interval reduction at two-month follow-up (e, f).

At the time of ophthalmic exam, the patient's visual acuity was 20/50 in the right eye and 20/60 in the left eye. Anterior segment examination demonstrated mild nuclear sclerotic cataracts and was notable for the absence of any signs of inflammation. Intraocular pressure by applanation tonometry was 15 in both eyes. Posterior segment examination revealed multiple foci of inner retinal opacification, or cotton wool spots, in the posterior pole of both eyes (Figures 1(a)–1(d)). Apart from these lesions, fundus examination was unremarkable without other signs of retinal vascular disease or inflammation. Despite the patient's history of hypertension, there was no narrowing of the retinal arterioles or arteriovenous crossing anomalies.

**Figure 2:**
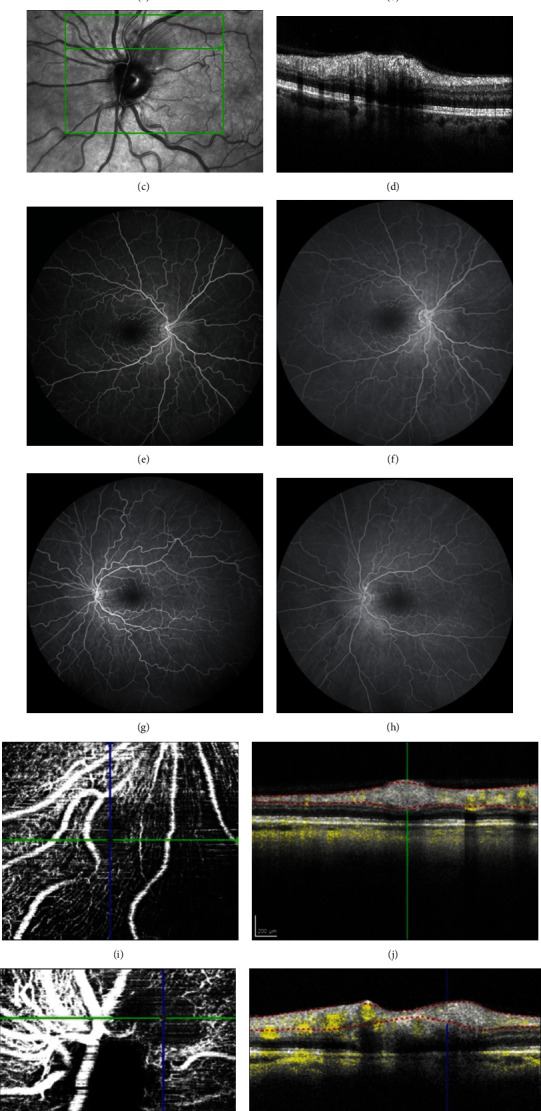
OCT demonstrated normal foveal contour in both eyes (a, b). OCT through an area of inner retinal opacification in the left eye (c, d) revealed thickening and hyperreflectivity of the nerve fiber layer. FA was largely unremarkable (e–h) aside from mild obscuration of the retinal vasculature in areas of inner retinal opacification. OCTA (i–l) sectioned at the superficial capillary plexus demonstrated flow voids corresponding to the cotton wool spots.

Optical coherence tomography (OCT) revealed no retinal edema (Figures 2(a) and 2(b)). OCT through the lesions revealed thickening and hyperreflectivity of the retinal nerve fiber layer (Figures 2(c) and 2(d)). Fluorescein angiography demonstrated mild obscuration of the retinal vasculature by the peripapillary cotton wool spot in the left eye; otherwise, the study was unremarkable (Figures 2(e)–2(h)). OCT angiography sectioned at the level of the superficial capillary plexus demonstrated flow voids corresponding to the location of the lesions (Figures 2(i)–2(l)).

The patient was counseled on his diagnosis and seen for follow-up two months later. Upon his return, the patient reported improved vision in both eyes. Visual acuity was 20/20 in the right eye and 20/25 in the left eye. On fundus examination (Figures 1(e)–1(f)), there was marked reduction in the size and number of cotton wool spots in both eyes.

## 3. Discussion

Our patient's clinical course, complicated by multiorgan failure, and ocular findings appear most consistent with a Purtscher-like retinopathy following severe COVID-19 illness. Retinal anomalies have been among the multitude of reports on the COVID-19 and the novel coronavirus pandemic [3–8]. SARS-CoV-2 has been detected in the retinas of COVID-19 patients on autopsy [4]. Case reports documenting retinal findings have described a variety of vasculopathic presentations, including papillophlebitis, acute ophthalmic artery occlusion, paracentral acute middle maculopathy, acute macular neuroretinopathy, and cotton wool spots [5–8].

In reporting on cotton wool spots in 6 of 27 patients recovered from COVID-19 pneumonia, Landecho et al. suggested that retinal microangiopathic changes may result from the hypercoagulable state seen in cases of COVID-19 or direct viral infection of the vascular endothelium leading to vasculitis [8]. Both of these proposed mechanisms of microangiopathic injury—coagulopathy and vasculitis—are also postulated to be involved in the pathogenesis of Purtscher's and Purtscher-like retinopathy. Microemboli to the retina are suspected to result in the constellation of posterior pole lesions in Purtscher-like retinopathy [1, 2]. The source of the microemboli—air, fat, leukocyte aggregates, platelets, and fibrin—is supposed to vary along with the causes of Purtscher-like retinopathy [2]. Vasculitis, induced by lipase or other inflammatory cytokines, may also lead to thrombosis and vascular occlusion resulting in Purtscher-like retinopathy [2].

Given these similarities in presentation and proposed pathogenesis, it seems appropriate to call the retinal microangiopathy seen in our COVID-19 patient a Purtscher-like retinopathy. One may question whether it is appropriate to ascribe our patient's retinopathy to his COVID-19 infection or to the multiorgan failure and coagulopathy that complicated his clinical course. In considering this question, the report of Landecho et al. is particularly useful, as their patients—whose retinal findings were also consistent with Purtscher-like retinopathy—did not have multiorgan failure or a clinical course as advanced as ours. One may also question if our patient's retinopathy is best described as a Purtscher-like retinopathy or is better attributed to his hypertension or his underlying diabetes. This is a reasonable concern as the diagnosis of Purtscher-like retinopathy is clinical and must consider a wide differential diagnosis. Indeed, our patient lacked the low to moderate number of intraretinal hemorrhages and pathognomonic Purtscher flecken of Purtscher-like retinopathy. However, given the severity of his systemic illness, our patient had a delayed ocular presentation and thus the acute findings of Purtscher-like retinopathy, hemorrhages, and Purtscher flecken may have previously resolved. Furthermore, aside from cotton wool spots, our patient demonstrated none of the characteristic findings of these retinopathies, though the possibility of a delayed presentation of a hypertensive crisis without chronic hypertensive changes remains.

## 4. Conclusions

In the weeks and months ahead, ophthalmologists will no doubt encounter more and more patients recuperating from COVID-19 infections. We call attention to the findings in our patient and suggest ophthalmologists to be attentive to this Purtscher-like retinopathy. Our observations of microangiopathy in these patients may well contribute to a more comprehensive understanding of the burden of COVID-19.

## Data Availability

The data used to support the findings of this report are included within the article.
